# Case Report: Type A invasive thymoma with renal metastasis

**DOI:** 10.3389/fonc.2025.1448753

**Published:** 2025-02-27

**Authors:** Xiao-Xia Wang, Xiao-Dong Zhao, Ming Wang

**Affiliations:** ^1^ Department of Oncology, Weihai Central Hospital Affiliated to Qingdao University, Weihai, Shandong, China; ^2^ Department of Gastroenterology, Weihai Central Hospital Affiliated to Qingdao University, Weihai, Shandong, China

**Keywords:** thymoma, renal metastasis, diagnosis, treatment, chemotherapy

## Abstract

Thymomas are rare malignancies originating from the epithelial cells of the thymus, typically presenting in the anterior mediastinum. Despite their indolent nature and slow growth, thymomas have the potential to metastasize to various organs, including the lungs, bones, and liver. However, renal metastasis is exceedingly rare. This case reported the details a 65-year-old male diagnosed with invasive thymoma (Type A, Masaoka stage IV) with renal metastasis seven years after the initial diagnosis. The patient underwent laparoscopic-assisted radical nephrectomy, followed by chemotherapy. The case demonstrated the potential for long-term survival among patients with advanced stages of thymoma when managed with a multidisciplinary approach, and underscored the need for monitoring extrathoracic metastases during the follow-up of patients with thymomas, thereby improving early detection and survival of these patients.

## 1 Introduction

Thymomas originate from epithelial cells of the thymus, typically located in the anterior mediastinum. It is a rare disease, accounting for only 0.2–1.5% of all malignancies (1). The incidence rates of thymoma were estimated to be approximately 0.22 per 100,000 person-years in the United States (2), 0.26 per 100,000 person-years in Europe (2), 0.29 per 100,000 person-years in Japan (3), and 0.40 per 100,000 person-years in China (4). Most patients with thymoma are aged between 40 and 60, with the incidence rate similar between men and women (2). Thymomas generally grow slowly, and their clinical symptoms are mainly attributed to the tumor’s increase in size, compressing surrounding tissues or organs. As the tumor grows, patients may experience more severe symptoms such as retrosternal pain, chest oppression, shortness of breath, and swelling of the head and face (5).

The WHO classification categorizes thymomas into several distinct types based on their histological features, such as the architecture and the predominant cell types involved. For instance, Type A thymomas are characterized by a predominance of spindle-shaped, neoplastic thymic epithelial cells with minimal cellularity, leading to a mostly indolent behavior and low invasiveness. In contrast, Type B thymomas, particularly B2 and B3, show a more aggressive behavior with a higher incidence of local invasion and metastasis. Type B2 thymomas exhibit a mix of lymphocyte and epithelial cells, while Type B3 thymomas demonstrate a predominance of epithelial cells, both of which contribute to a more aggressive clinical course. In addition, Type A and AB thymomas are associated with better overall survival and lower recurrence rates, while Type B2 and B3 thymomas tend to have poorer outcomes due to their increased invasiveness and likelihood of recurrence.

Thymomas often involve surrounding structures. However, extrathoracic metastases are fairly rare (6), with an estimated prevalence ranging from 3% to 6% (7, 8). Only a limited number of reports confirmed their metastatic potential to distant sites, such as the brain (9), kidneys (8), breasts (10), extrathoracic lymph nodes (11), liver (6), lung (12), and bones (13). Although renal metastasis is quite rare, it does exist (8). Here, we reported a case of thymoma with kidney metastasis, aiming to improve our understanding on the metastatic characteristics of thymomas and provide references for the treatments of thymomas in clinical practice.

## 2 Case description

A 65-year-old male patient was admitted to the hospital on February 17, 2016, presenting with chest oppression and chest pain that had persisted for one month. An enhanced chest CT conducted upon admission revealed a mass in the anterior mediastinum measuring approximately 12.5 cm x 5.0 cm, highly suggestive of an invasive thymoma, with accompanying pericardial and bilateral pleural effusions. Subsequently, on February 22, 2016, the patient underwent drainage of the pericardial effusion under local anesthesia, along with an ultrasound-guided biopsy of the suspected thymic tumor. Results of pathological examination confirmed the presence of a thymoma, with a high likelihood of type A. Immunohistochemical analysis provided the following results: CK-pan (3+), Vimentin (-), CK19 (3+), CK5/6 (3+), P63 (3+), CD20 (-), CD56 (-), Syn (-), Cga (-), TTF1 (-), CD5 (-), CD117 (-), Ki67 (+5-10%) (**Table 1**). In addition, the reticular fiber staining was positive. Based on these findings, the patient received an initial diagnosis of invasive thymoma (Type A, Masaoka stage IV) with pericardial and pleural metastases.

**Table 1:** Immunohistochemical analysis results of tumor biomarkers at different time points.

Parameters	Feb 22, 2016	May 26, 2023	Jul 22, 2023
CK-pan	3+	+	N/A
P63	3+	+	+
CK5/6	3+	N/A	N/A
Vimentin	–	–	N/A
CK19	3+	N/A	N/A
CD20	–	–	N/A
CD56	–	–	N/A
Syn	–	–	N/A
Cga	–	N/A	N/A
TTF-1	–	N/A	N/A
CD5	–	N/A	Scattered +
CD117	–	N/A	–
Ki-67	5-10% +	5%+	10%+
PAX8	N/A	–	Partially weak +
CD10	N/A	–	N/A
CD34	N/A	–	N/A
Bcl-2	N/A	+	N/A
CD99	N/A	–	N/A
GATA-3	N/A	N/A	–
CK7	N/A	N/A	+
CK20	N/A	N/A	–
TdT	N/A	N/A	–

N/A, Not Applicable; CK-pan, Cytokeratin - pan; CK5/6, Cytokeratin 5/6; CK19, Cytokeratin 19; CD20, Cluster of Differentiation 20; CD56, Cluster of Differentiation 56; Syn, Synaptophysin; CgA, Chromogranin A; TTF-1, Thyroid Transcription Factor-1; CD5, Cluster of Differentiation 5; CD117, Cluster of Differentiation 117; PAX8, Paired Box Gene 8; CD10, Cluster of Differentiation 10; CD34, Cluster of Differentiation 34; Bcl-2, Bcell lymphoma 2; CD99, Cluster of Differentiation 99; GATA-3, GATA-binding protein 3; CK7, Cytokeratin 7; CK20, Cytokeratin 20; TdT, Terminal deoxynucleotidyl transferase.

On March 1, 2016, the patient received a chemotherapy regimen of docetaxel 120mg on day 1 plus carboplatin 400mg on day 2, every 3 weeks (q3w). Subsequently, he underwent palliative radiotherapy on April 25, 2016, aimed at alleviating symptoms and controlling the tumor growth. The prescribed doses were 59.4 Gy for the planning gross tumor volume (PGTV) and 54 Gy for the planning target volume (PTV), delivered in 27 fractions. Upon the completion of radiotherapy, the patient was re-admitted on July 18, 2016, for two additional cycles of chemotherapy (docetaxel 120mg on day 1 plus nedaplatin 60mg on days 1-2, q3w). Routine follow-ups were scheduled, during which an examination on November 24, 2016 indicated disease progression. Consequently, the patient initiated a monotherapy regimen on the following day, consisting of 60mg of tegafur, administered orally twice a day (bid) on days 1-14 of a 21-day cycle, for a total of three cycles.

A chest CT scan was performed on July 24, 2020, and revealed the presence of nodules in the thymic area and multiple nodules in both lungs, suggesting the possibility of recurrence and metastasis. Subsequently, the patient underwent 4 cycles of chemotherapy on July 25, August 14, September 4, and September 28, 2020, consisting of docetaxel 140mg on day 1 plus nedaplatin 70mg on days 1-2, every 3 weeks.

On May 24, 2023, the patient was readmitted to the hospital due to the discovery of a lump in the lower left abdomen for a week. Subsequently, an enhanced CT of the chest and abdomen was performed, revealing an abnormally enhanced mass in the anterior mediastinum with a maximum cross-sectional area of approximately 8.3 cm x 2.2 cm, along with multiple moderately enhanced nodules and masses of varying sizes in both lungs, suggesting the presence of metastatic tumors. In addition, a significant space-occupying lesion was observed in the left kidney, suspected to be malignant. On May 26, 2023, a biopsy of the mass in the left kidney was carried out, and exhibited perivascular arrangement of both epithelioid and short spindle-shaped tumor cells, with occasional mitotic figures observed. Results of immunohistochemistry were as follows: PAX8 (-), CD10 (-), CK (+), P63 (+), Ki-67 (+, 5%), CD20 (-), CD34 (-), Bcl-2 (+), CD56 (-), Syn (-), Vimentin (-), CD99 (-) (**Table 1**).

Based on the aforementioned findings and medical history, the patient was finally diagnosed as invasive thymoma (Type A, Masaoka stage IV), accompanied with pericardial metastasis, pleural metastasis, bilateral lung metastasis, and renal metastasis.

Following the multidisciplinary team (MDT) consultation, a treatment regimen was formed, with a primary emphasis on chemotherapy. Meanwhile, surgical intervention was also considered due to the obvious left kidney metastasis. The patient underwent two cycles of chemotherapy consisting of docetaxel (120mg on day 1) and nedaplatin (140mg on day 2), administered every 3 weeks.

**Figure 1:**
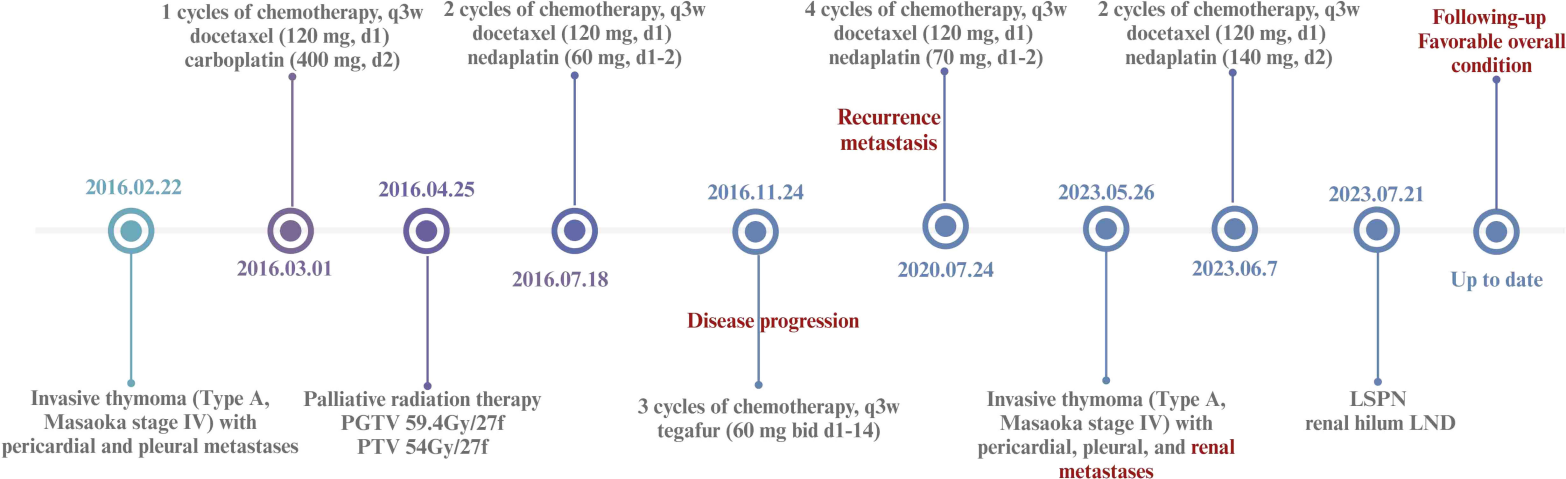
A timeline illustration summarizing the diagnosis and treatment pathway of the case.

**Figure 2:**
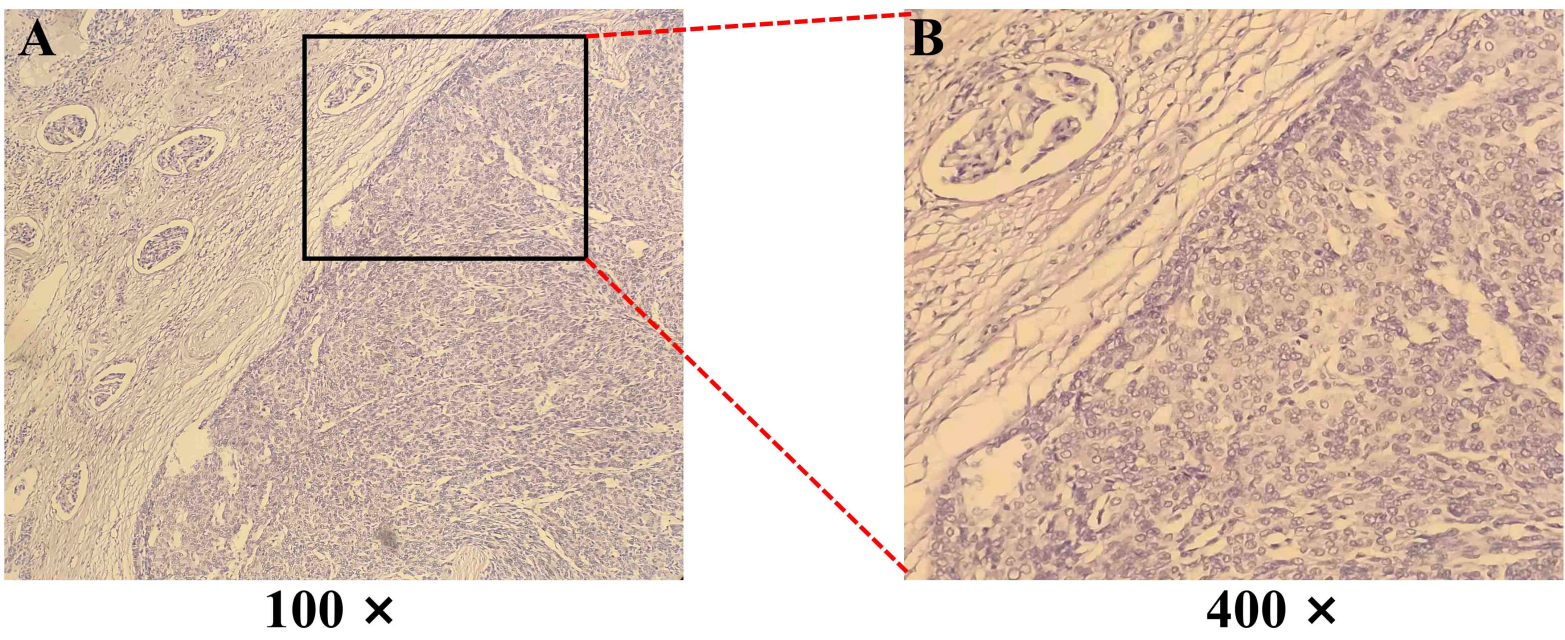
Hematoxylin-eosin (H&E) staining of a biopsy from a Thymoma (Type A) with renal metastasis. **(A, B)** The tumor cells exhibit an epithelioid, short spindle shape, arranged in sheets and perivascular patterns. Magnification: 100× and 400×.

**Figure 3:**
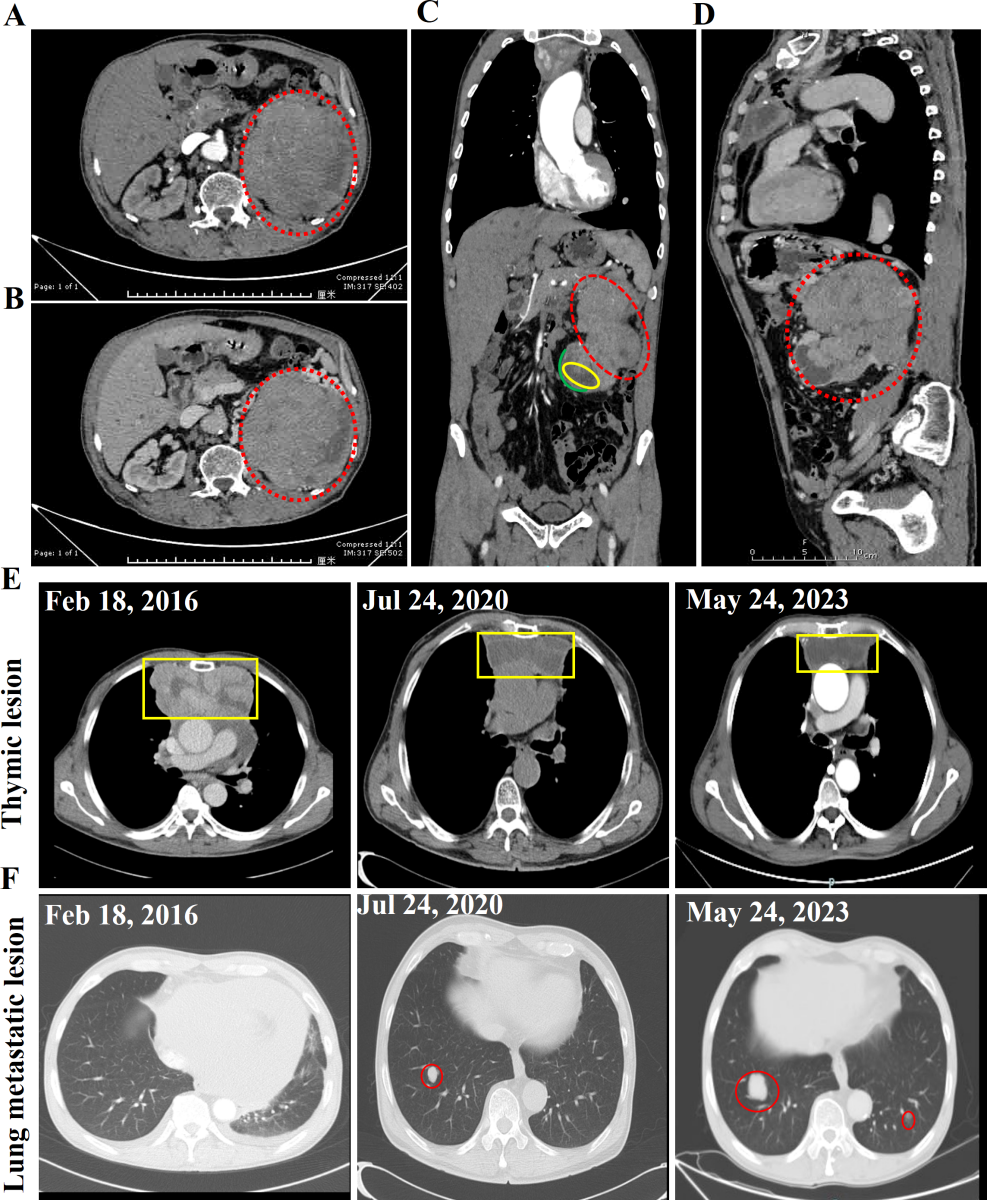
Abdominal contrast-enhanced CT scan images of this case. The normal morphology of the left kidney is absent, and a large, heterogeneously enhancing mass is observed, with the degree of enhancement being most prominent in the venous phase. The largest cross-sectional area of the lesion measures approximately 12 cm × 11 cm, and multiple tortuous vascular shadows are seen surrounding the lesion. **(A)** Arterial phase. **(B)** Venous phase. **(C)** Coronal planes. **(D)** Sagittal plane. The dotted red line indicates the thymoma renal metastasis tumor. The yellow box highlights the hydronephrosis, while the green line delineates the renal cortex. **(E)** Enhanced CT images of the thymic lesion **(E)** and lung metastatic lesion **(F)** at different time points. The yellow box indicates the thymoma lesion, while the red circle denotes the lung metastatic lesion.

Next, the patient underwent laparoscopic-assisted radical left nephrectomy and renal hilum lymph node dissection under general anesthesia on July 22, 2023 (**Figure 1**). Results of immunohistochemistry showed P63(+), CD5(scattered+), CD117(-), Ki-67(+,10%), PAX8(partially weak+), GATA-3(-), CK7(+), CK20(-), TdT(-) (**Table 1**). Postoperative pathological examination further confirmed the diagnosis of atypical thymoma (Type A) with metastasis to the kidney (**Figure 2**), which was consistent with the findings obtained from immunohistochemistry and in accordance with his medical history. The tumor size reached up to 16cm×10 cm (**Figure 3**). No tumor was found at the vascular and ureteral stumps. However, infiltration of the surrounding adipose capsule and renal sinus was observed. A tumor thrombus was identified in the renal vein. No evidence of tumor invasion was detected in the adrenal gland, and no tumor was found in the lymph nodes at the left renal hilum (0/6). The patient was kept under close follow-up and his overall condition remained favorable after the surgery.

## 3 Discussion

Thymoma is the most common primary tumor of the anterior mediastinum, accounting for about 20% of all mediastinal tumors (5). As a relatively indolent tumor, some patients with thymoma can survival for a long time even after disease progression and recurrence, with a 5-year survival rate approaching 90% (14). In this case, the patient was initially diagnosed with invasive thymoma in 2016, accompanied with pericardial and pleural metastases. Thus, a comprehensive treatment, including radiotherapy and chemotherapy, was administered. However, lung metastasis was discovered in 2020, leading to further chemotherapy. Unfortunately, seven years following the initial diagnosis, kidney metastasis was also detected, necessitating both surgical intervention and chemotherapy.

When determining the treatment for thymoma, several factors need to be taken into consideration, including the tumor’s location, extent of invasion, and staging. Based on these factors, appropriate treatment methods can be selected, which may include surgery, radiotherapy, chemotherapy, and ablation. If there is no distant metastasis and the tumor is completely resectable, surgery would be the preferred option. Patients who underwent complete resection have better survival and recurrence rates than those who have a partial resection, simple biopsy, or inoperable tumor (15). Among all thymomas, 35-40% are locally advanced (Masaoka stages III-IV) or metastatic, often considered only partially resectable, or not resectable at all (16), necessitating induction therapy. Induction therapy primarily consists of chemotherapy alone and concurrent chemoradiotherapy, with radiotherapy alone remaining controversial on optimal circumstance under which it should be administered (17, 18). The potential advantages of induction therapy include tumor down-staging, increased likelihood of an R0 resection, and prevention of systemic progression (19, 20). Platinum-based combination chemotherapy regimens are commonly utilized in the treatment of thymoma. Kao TN et al. conducted a retrospective analysis involving 30 patients with advanced thymomas who received induction chemotherapy or chemoradiotherapy before tumor resection, and the results showed that in 40.0% of the patients, the tumor size decreased after induction therapy, and 70% achieved R0 resection (16). In this case, the patient was diagnosed with invasive thymoma (Type A, Masaoka stage IV), accompanied by pericardial metastasis, pleural metastasis, bilateral lung metastasis, and renal metastasis. Given the presence of multiple metastases and the advanced stage of the tumor, surgical intervention was not deemed the optimal treatment approach. Therefore, comprehensive treatment was implemented, primarily consisting of radiotherapy and chemotherapy.

Thymomas primarily exhibit local infiltration but can also present with intrathoracic recurrence and even distant metastasis, a biological characteristic of the disease. The most common site for distant metastasis is the lungs (12, 21). Metastases to the kidney are rare and were historically described in autopsy series, and the incidence ranged between 2.36% and 12.6% (22). In this case, a significant occupying lesion in the left kidney was discovered seven years after the initial diagnosis of thymoma. Based on the results of immunohistochemistry, imaging and medical history, it was confirmed as a thymoma with metastasis to the kidney. Most renal metastases originate from the hematogenous spread of the primary tumor, likely due to the rich blood supply to the kidneys, which facilitates the hematogenous spread of tumor cells (15). The primary clinical manifestations of secondary renal tumors usually include back pain, gross hematuria, and palpable abdominal mass; However, most cases are asymptomatic, and the tumor is usually found incidentally on imaging examination (23). Due to the nonspecific clinical presentation, it may be misdiagnosed as other tumors and needs to be differentiated from urothelial carcinoma, primary or metastatic squamous cell carcinoma, synovial sarcoma, and other tumors (22). Secondary tumors of the kidney generally represent an advanced stage of the primary tumor, with a poor prognosis. Due to the diversity of primary tumors, there is no unified treatment strategy for secondary renal tumors, and they are commonly treated with a comprehensive approach based on the late stage of the primary tumor. In this case, MDT consultation was adopted and a treatment regimen was formed. The patient in this case report received the chemotherapy, followed by left radical nephrectomy, which effectively alleviated his symptoms such as back pain and hematuria.

Due to its rarity of thymoma with renal metastasis, MDT consultation was of great significance in formulating a comprehensive treatment plan for this patient. The complexity of managing advanced stages of thymoma, especially when coupled with rare metastatic patterns, necessitates a collaborative approach that leverages the diverse expertise of various specialists, including medical oncologists, surgical oncologists, radiologists, pathologists, and other relevant specialists (24). They serve as a critical platform for discussing various management aspects of cancer cases, ensuring that patients receive state-of-the-art care that is both evidence-based and patient-centered (25). In the context of this case, the MDT consultation was pivotal in deciding the sequence and combination of chemotherapy and surgical intervention. The decision to proceed with laparoscopic-assisted radical nephrectomy following chemotherapy was informed by the collective insights of the team, taking into account the patient’s overall condition, the extent of the disease, and the potential for long-term survival.

The pathological classification and clinical staging have been found to be significantly associated with the prognosis of patients with thymomas (26). Based on the presence or absence of a capsule, thymoma can be classified into non-invasive and invasive types. Meanwhile, according to pathology and prognosis, tumors can be divided into a low-risk group (types A, AB, and B1), a high-risk group (types B2 and B3), and a thymic carcinoma group (type C). The fifth edition of the WHO classification of thymic epithelial tumors (revised in 2021) (27) recommended the thymomas to be classified as type A thymoma (including an atypical variant), AB thymoma, type B thymoma (separated into B1, B2, and B3 thymomas), micronodular thymoma with lymphoid stroma, and metaplastic thymoma by histologic features and immunohistochemistry, with type C eliminated. Besides, thymic carcinoma is listed as a separate category independent of thymoma. There are various schemes for the clinical staging of thymoma, among which Masaoka staging system was the most prevalent. This system categorizes thymomas based on their extent of local invasion and involvement of surrounding structures, consisting of four main stages: I, II, III, and IV (28). There is a close correlation between the Masaoka staging system and tumor prognosis (26). A study conducted in Germany (29) involving 228 patients revealed no tumor-related deaths among patients with types A, AB, or B1 thymoma at Masaoka stages I and II; However, 50-60% of the patients with type B2, B3 thymoma, or thymic carcinoma at Masaoka stages III-IV exhibited tumor-caused mortality rates of 9%, 19%, and 17%, respectively. Based on the above research results, it could be speculated that the long-term prognosis of this case may be poor.

In summary, for advanced stages of thymoma, radiotherapy and/or chemotherapy should be considered, combined with local ablation or surgical interventions when necessary. Moreover, during the follow up of patients with thymoma, attention should be paid to the possibility of metastatic tumors in the organs such as the kidneys, in addition to the lung. Early detection of extrathoracic metastases and timely treatment of these patients can improve the prognosis of the patients. Further multicenter and prospective clinical trials with diverse study populations will be necessary to provide a more comprehensive understanding of this condition, and to establish a clear consensus of treatment guidelines for locally advanced thymomas.

## Data Availability

The original contributions presented in the study are included in the article/supplementary material. Further inquiries can be directed to the corresponding author.
